# Synergistic immunotherapy targeting cancer-associated anemia: prospects of a combination strategy

**DOI:** 10.1186/s12964-023-01145-w

**Published:** 2023-05-19

**Authors:** Ting Yuan, Qingzhu Jia, Bo Zhu, Degao Chen, Haixia Long

**Affiliations:** 1 Institute of Cancer, Xinqiao Hospital, Third Military Medical University, Chongqing, 400037 China; 2 Chongqing Key Laboratory of Immunotherapy, Xinqiao Hospital, Third Military Medical University, Chongqing, 400037 China

**Keywords:** Cancer-associated anemia, Erythropoiesis, Extramedullary hematopoiesis, Eryptosis, Immune checkpoint inhibitors

## Abstract

Cancer-associated anemia promotes tumor progression, leads to poor quality of life in patients with cancer, and even obstructs the efficacy of immune checkpoint inhibitors therapy. However, the precise mechanism for cancer-associated anemia remains unknown and the feasible strategy to target cancer-associated anemia synergizing immunotherapy needs to be clarified. Here, we review the possible mechanisms of cancer-induced anemia regarding decreased erythropoiesis and increased erythrocyte destruction, and cancer treatment-induced anemia. Moreover, we summarize the current paradigm for cancer-associated anemia treatment. Finally, we propose some prospective paradigms to slow down cancer-associated anemia and synergistic the efficacy of immunotherapy.

Video Abstract

**Supplementary Information:**

The online version contains supplementary material available at 10.1186/s12964-023-01145-w.

## Introduction

Anemia is one of the most common complications in patients with cancer, and cancer-associated anemia is indicative of a poor prognosis, irrespective of tumor type. The incidence of anemia in patients with cancer varies according to cancer type, stage, and therapeutic intervention [1, 2]. According to World Health Organization (WHO) criteria and the Common Terminology Criteria for Adverse Events (CTCAE) (v 5.0), cancer-associated anemia is defined as a hemoglobin (Hb) level lower than 120 g/L in women and lower than 130 g/L in men [3]. Anemia in patients with cancer can be classified as cancer-induced anemia, which affects approximately 40% of patients newly diagnosed with cancer and prior to any antineoplastic treatment; and cancer treatment-induced anemia, which affects approximately 40%–70% of patients who are not anemic at diagnosis but develop anemia as a universal side effect of the cancer therapy [2, 4, 5]. The underlying mechanisms of cancer-induced anemia include decreased erythropoiesis and increased erythrocyte destruction, and strategies for treating cancer-induced anemia have been established based on these mechanisms.

**Fig. 1:**
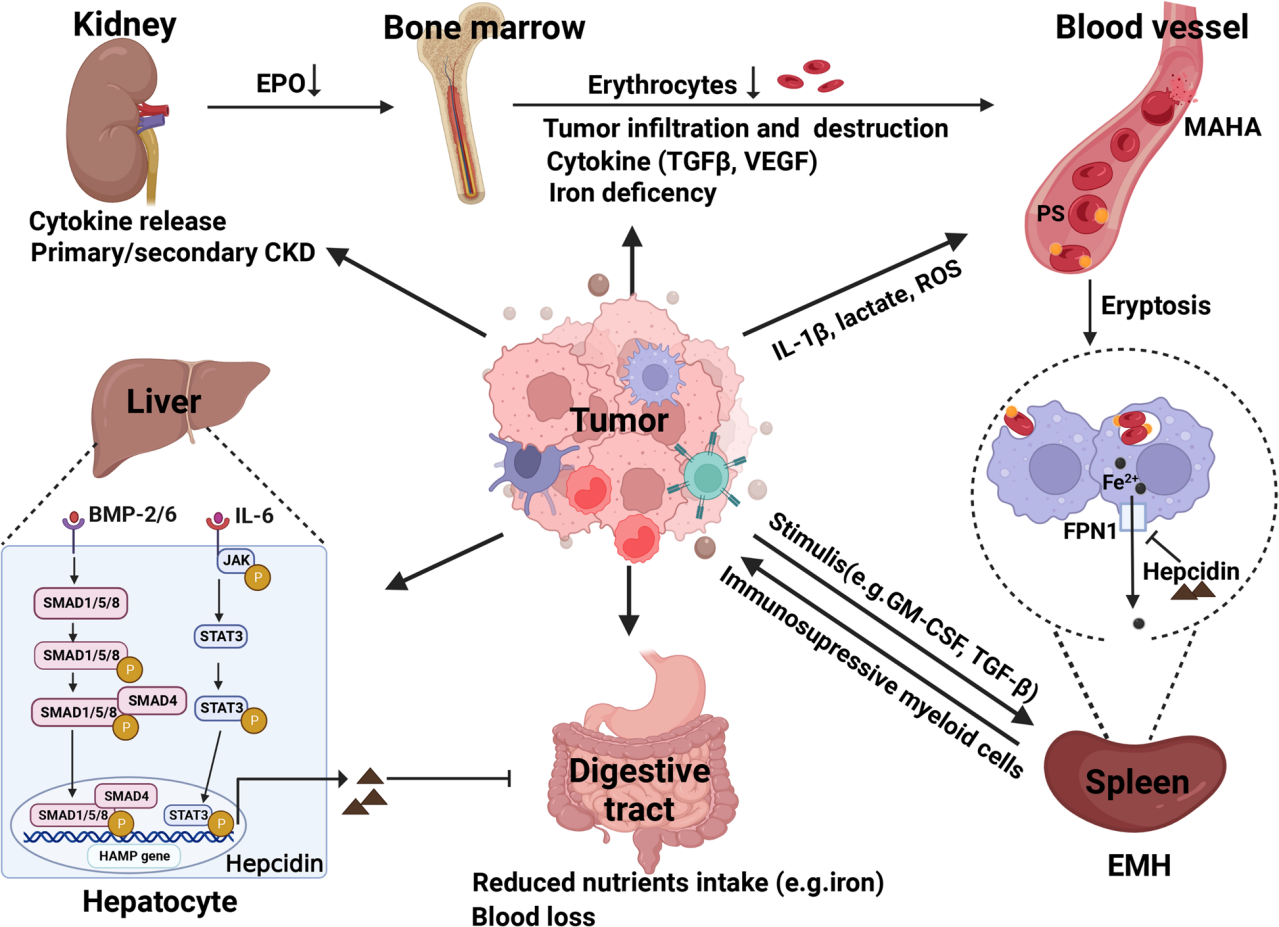
Mechanisms of cancer-induced anemia. Non-hematopoietic tumors can cause anemia via a range of mechanisms. Tumor-produced cytokines inhibit erythropoiesis directly or indirectly by blunting EPO production and inducing state of iron deficiency. Decreased EPO production is also common in cancer patients with the primary or secondary chronic kidney disease (CKD). Hepcidin, the key regulator of iron metabolism, inhibits iron flux in both gastrointestinal tract and splenic macrophages, and it is upregulated mainly by IL-6/STAT3 and BMP/SMAD pathway. Tumor-released stimulators, including GM-CSF and TGF-β, also trigger extramedullary hematopoiesis (EMH), which causes the defective erythropoiesis and induces immunosuppressive myeloid cells to accelerate tumor progression. Tumor infiltration of bone marrow results in hematopoietic environmental destruction and gastrointestinal tumor progression often accompanied by blood loss. Tumor-released IL-1β, lactate and ROS aggravate the process of eryptosis to promote erythrocytes destruction

Although the anti-cancer efficacy of immune checkpoint inhibitors (ICIs) has allowed immunotherapy to become an established cancer therapy, a long-term clinical response and increased overall survival is only seen in a subset of patients with specific cancer types [6]. In addition to several biomarkers, including programmed cell death ligand-1 (PD-L1) [7], tumor mutational burden (TMB) [8], DNA damage repair [9], and tertiary lymphoid structures [10] used to predict ICIs efficacy, cancer-associated anemia has been identified as a risk factor that reduces ICIs efficacy [11, 12]. As approximately 40% of patients with cancer have cancer-induced anemia, elucidating the effect of cancer-induced anemia on ICIs treatment efficacy, and developing strategies to simultaneously treat cancer and anemia is of significant clinical interest. Here we review recent research regarding the mechanism (Fig. 1) and treatment of cancer-induced anemia, and highlight the prospective paradigm of immunotherapy for cancer-induced anemia.

### Mechanisms of cancer-induced anemia

#### Influence of tumor-induced cytokines on erythropoiesis

Normal erythropoiesis is a continuous process occurring in the bone marrow. Early erythroid progenitor cell (EPC)-burst-forming unit-erythroid (BFU-E) are generated in stem- and progenitor cell niches and migrate to erythroblastic islands where the BFU-E differentiate into erythropoietin (EPO)-dependent erythroid progenitors and terminal erythroblasts. Erythroblasts then undergo hemoglobinization, enucleation, and subsequently enter the blood circulation to maintain erythrocyte mass and meet oxygen requirements. The proliferative and differentiative capacity of EPCs is sensitive to stimulatory or inhibitory factors [2], such as transforming growth factor-β (TGF-β) and vascular endothelial growth factor (VEGF).

In normal bone marrow, hematopoietic stem cell (HSC) quiescence and self-renewal is regulated by TGF-β [13]. In vitro, TGF-β plays a paradoxical role in erythropoiesis by blocking proliferation while accelerating the differentiation of erythroid progenitors [14]. Tumor-secreted TGF-β has been shown to inhibit erythropoiesis via organ-specific mechanisms. In advance-stage cancer, especially hepatocellular carcinoma [15], tumor-secreted TGF-β accelerates the differentiation of megakaryocytes and erythroid progenitors into splenic CD45^−^EPCs [16], which blocks the late stage of erythropoiesis. TGF-β knockout in hepatocellular carcinoma cells and specific-antibody TGF-β neutralization has been shown to reduce the number of splenic CD45^−^EPCs in vivo [16]. Furthermore, a study using Lewis lung carcinoma cells to generate a cancer-induced anemia model found that TGF-β secreted by osteoclasts induces deterioration of the HSC niche in the bone marrow. Treatment with a TGF-β inhibitor therefore improves erythropoiesis and ameliorates cancer-induced anemia [17].

Cancer-induced anemia is also associated with elevated plasma levels of VEGF [18, 19]. VEGF produced by tumor and stromal cells induces neovascularization, vessel remodeling, and expansion of early-stage EPCs in the bone marrow and extramedullary region [20], as well as vascular dilation and bone marrow cell mobilization, which induce HSC depletion through VEGF-VEGFR2 signaling [21]. Bevacizumab, an anti-VEGF antibody, reverses VEGF-induced severe anemia and reduces mortality in tumor-bearing mice [22], indicating that VEGF may be a promising target for cancer-induced anemia treatment.

#### Impaired systemic iron metabolism

Maturation of erythroid progenitors into erythrocytes is a multi-stage, iron-dependent process. Transferrin, which binds free iron with high affinity, attaches to EPC transferrin receptors. In acidified lysosomes of EPCs, iron is released from transferrin and exported to the cytoplasm by the divalent metal transporter 1 (DMT1). The iron subsequently enters the mitochondria and attaches to protoporphyrin IX during the final step of heme biosynthesis [23]. Cancer-induced anemia is commonly characterized by decreased serum iron concentrations and transferrin saturation despite sufficient iron stores, known as functional iron deficiency anemia (FIDA) [24, 25]. Furthermore, absolute iron deficiency anemia (AIDA) may be triggered by, for example, advanced gastrointestinal cancer, which is accompanied by reduced iron intake, defective iron absorption, and chronic blood loss [24, 26].

Systemic iron metabolism is regulated by hepcidin, which is mainly derived from the liver. Hepcidin is also produced by inflammatory monocytes or macrophages through a toll-like receptor (TLR)-4-dependent pathway in mice or an interleukin (IL)-6-dependent pathway in humans [27]. Increased hepcidin inhibits DMT1 and duodenal cytochrome b, thereby inhibiting intestinal iron absorption [28]. Hepcidin also binds to iron transporters on macrophages and duodenal cells, promoting internalization and degradation of ferroportin (FPN), which inhibits iron output and utilization [29, 30]. The consequent iron retention leads to insufficient plasma iron levels for erythropoiesis, resulting in FIDA [31]. Overexpression of hepcidin and FIDA are often observed in cancer patients [32], and tumor-produced hepcidin has been shown to reduce serum iron levels and cause severe anemia in a mouse model [33]. Several mediators, including IL-6, bone morphogenetic protein (BMP)-2, and growth differentiation factor-15 (GDF-15), regulate hepcidin levels.

IL-6, a proinflammatory cytokine, has been associated with cancer-induced anemia. In patients with renal cell carcinoma, IL-6 levels greater than 10 pg/mL were associated with a marked increase in the risk of anemia [34]. In patients with advanced ovarian cancer, serum IL-6 levels were inversely correlated with Hb levels, and high serum IL-6 levels were attributed to M1 tumor-associated macrophages from the tumor microenvironment [35]. Moreover, cancer treatment with recombinant human IL-6 has been found to induced anemia and hypoferremia [36], and a cancer-induced anemia mouse model has been established by inoculation with IL-6-producing human or mouse tumor cell lines [37]. It has been suggested that high IL-6 plasma levels induce hypoferremia by upregulating hepatic hepcidin expression via signal transducer and activator of transcription 3 (STAT3) activation [38], as well as increasing plasma volume to promote anemia [39]. Treatment with an anti-IL-6 receptor (IL-6R) antibody reversed cancer-induced anemia [37].

In patients with multiple myeloma, BMP-2, rather than IL-6, has been suggested as the major inducer of hepcidin, with BMP-2 levels inversely correlated with Hb levels [40]. Hepcidin is also upregulated by BMP-6/small-mothers-against-decapentaplegic (SMAD) signaling, which is negatively correlated with Hb concentrations in patients with solid tumors [41, 42]. In addition, in patients with gastrointestinal cancer, hepcidin production is downregulated by increased GDF-15, mainly due to chronic blood loss [43]. These studies suggest that hepcidin inhibition may help alleviate cancer-induced anemia.

#### Reduced EPO production

EPO, a crucial cytokine regulator of erythropoiesis, is produced by kidney interstitial cells and is involved in the regulation of erythroid cell survival, proliferation, and differentiation. EPO binds to the EPO receptor (EPOR), which is expressed in erythroid cell stages from the colony-forming unit-erythroid (CFU-E) to the basophilic erythroblast, activating the JAK2-STAT1/3/5 [44] and PI3K-AKT-GATA-1 [45] pathways to regulate erythropoiesis. In addition, EPO-induced growth arrest-specific gene 6, released by erythroblasts, improves cell survival and maturation via autocrine or paracrine pathways [46]. However, compared with patients with iron-deficiency anemia, EPO production is reduced in patients with cancer-induced anemia [47, 48]. Mechanistically, EPO-deficiency could be attributed to inflammatory or immune responses involving cytokine release, as well as primary or secondary chronic kidney disease in patients with cancer [2, 49]. Moreover, increased intracellular iron sequestration may promote hypoxia inducible factor-1α (HIF-1α) degradation via prolyl hydroxylation, thereby reducing EPO production in patients with cancer [50]. Thus, EPO deficiency may contribute to anemia in patients with cancer.

#### Cancer-induced extramedullary hematopoiesis

**Fig. 2:**
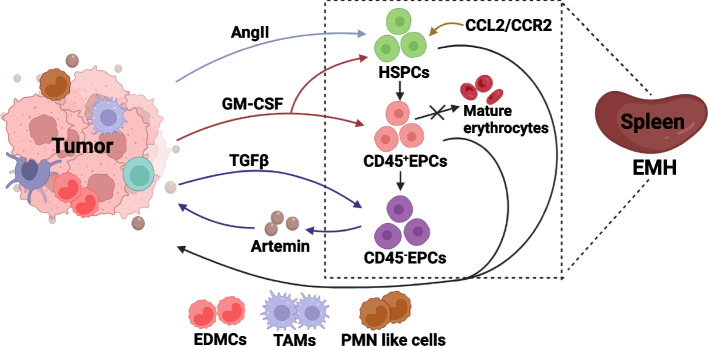
EMH and erythro-myeloid transdifferentiation during cancer-induced anemia. Tumor-derived stimulators, including AngII, GM-CSF and TGF-β, induce EMH. AngII and CCL2/CCR2 signal lead to the amplification of HSPCs in spleen and erythro-myeloid transdifferentiation as well. AngII triggers the sustained tumor associated macrophages (TAMs), and GM-CSF drives HSPCs into immunosuppressive PMN like cells. EPCs' differentiation is blunted in EMH, CD45^+^EPCs are incline to convert into EDMCs by the stimulation of GM-CSF, while CD45^−^EPCs accumulation induced by TGF-β produce artemin. All of which aggravates cancer-induced anemia directly or indirectly by tumor progression

In advanced cancer, extramedullary hematopoiesis is initiated to maintain erythroid homeostasis [51]. However, extramedullary hematopoiesis may cause deficient or ineffective erythropoiesis, leading to increased numbers of immature erythroid cells and a heightened splenic myeloid response [52] (Fig. 2). Tumor-derived VEGF [53] and platelet-derived growth factor (PDGF)-BB [54] induce extramedullary hematopoiesis, resulting in splenomegaly and tumor progression. Additionally, splenic macrophage-synthesized BMP-4 has been shown to promote splenic erythropoiesis in a 4T1 tumor model [55]. Recently, splenic immature erythroid cells were identified as EPCs (CD71^+^TER119^+^). In patients with cancer, abundant CD45^+^EPCs are accumulated in the spleen, producing reactive oxygen species (ROS), which inhibits systemic anti-tumor immunity, and CD45^+^EPCs promote tumor progression through CD8 T-cell immunosuppression [56]. In hepatic cancer, tumor-derived TGF-β contributes to the generation of splenic CD45^−^EPCs [16], which promote tumor progression via the neurotrophic factor artemin.

Tumor-induced extramedullary hematopoiesis also leads to erythro-myeloid transdifferentiation (Fig. 2). Melanoma-derived IL-3 skews erythroid hematopoiesis towards myeloid lineages, which are characterized by increased myeloid progenitors, specifically CMPs, GMPs, and MEPs, and decreased late-stage splenic erythroblasts [57]. In a mouse model of lung adenocarcinoma, overproduction of angiotensin II (Ang II) induced splenic HSCs and macrophage progenitor amplification, thus providing sustained tumor-associated macrophages during cancer progression [58]. Hepatic cancer-induced CCL2/CCR2 signaling and endogenous granulocyte–macrophage colony-stimulating factor (GM-CSF) have also been shown to drive differentiation of splenic HSCs into immunosuppressive myeloid cells-polymorphonuclear (PMN)-like cells [59]. In breast cancer, 4T1 tumors express granulocyte-CSF (G-CSF) to promote splenic myelopoiesis, causing anemia [60]. Furthermore, tumor-expressed GM-CSF stimulates CD45^+^EPC differentiation into erythroid differentiated myeloid cells (EDMCs) with an immunosuppressive phenotype, which reduces immune checkpoint therapy efficacy [11]. Tumor-derived growth factors and cytokines may therefore induce EPC accumulation in extramedullary organs, driving erythro-myeloid transdifferentiation into tumor tissue. And observational study suggested that intratumoral CD45^+^CD71^+^ erythroid cells suppressed T cells, predicted disease-free survival and overall survival in hepatocellular carcinoma [61]. Thus, further study of erythroid-derived immune cells in tumor tissue may provide new ideas for the outcome of extramedullary hematopoiesis and new targets for tumor therapy.

#### Increased erythrocyte destruction

Erythrocytes undergo eryptosis, a suicidal cell death characterized by cell shrinkage and cell membrane scrambling resulting in phosphatidylserine (PS) exposure. Binding of the exposed PS to its receptors on red pulp macrophages (RPMs) in the spleen and liver induces rapid clearance of circulating erythrocytes [62]. In tumor-bearing hosts, sustained erythrocyte elimination leads to anemia [63]. Eryptosis plays an important role in accelerating anemia and although numerous mechanisms of eryptosis mediation have been reported for various pathological conditions, the mechanism of cancer-induced eryptosis is not well understood (Fig. 1). Patients with cancer exhibit two to three times greater PS-positive erythrocyte levels than healthy individuals [63, 64], and tumor-induced inflammatory and metabolic remodeling has been shown to increase PS-positive erythrocytes and augment splenic phagocyte activity in tumor-bearing mice [65]. Specifically, IL-1β, propionylcarnitine and valerylcarnitine, as well as TG (54:4) increases PS externalization on erythrocytes, and PS externalization on erythrocytes is induced via the IL-1β/IL-1 receptor 1/caspase 3 pathway. IL-1β and lactate additionally promote the phagocytic activity of splenocytes, which subsequently engulf the erythrocytes [65]. In addition to cytokines and metabolites, increased ROS production may induce eryptosis. Clinical data have shown significantly increased erythrocytic ROS production in patients with lung cancer [63], with the ROS subsequently inducing eryptosis by impairing cytoskeletal proteins [66]. Further studies of the mechanism of tumor-cell induced eryptosis in patients with cancer-induced anemia is, however, required.

Cancer-related microangiopathic hemolytic anemia (MAHA) involves the excessive destruction of erythrocytes and is characterized by the presence of erythrocyte fragments or schistocytes in small blood vessels. It is a serious complication in cancer and is associated with cancer recurrence, metastasis, and a poor prognosis [67]. Cancer can cause MAHA through systemic microvascular metastases, bone marrow metastases, or tumor necrosis. Erythrocyte fragmentation may result from direct contact with tumor emboli or intraluminal fibrin thrombi within blood vessels, and hypersplenism [68–70]. Furthermore, bone metastases may lead to secondary myelofibrosis, inducing direct release of prothrombotic ultra-large von Willenbrand factor multimers and promoting platelet aggregation [71, 72]. Massive tumor necrosis also induces tissue-factor production, which initiates the coagulation cascade and leads to thrombotic microangiopathies [73].

In addition to MAHA, autoimmune hemolytic anemia (AIHA) is a paraneoplastic phenomenon that occurs in most types of solid tumors, including lung, colorectal, renal, and ovarian cancers [74]. Secondary warm-antibody AIHA, the most prominent type of AIHA, develops as a result of tumor- or therapy-induced production of IgG, IgM, or IgA, which bind to the erythrocyte surface and enhance erythrocyte trapping and phagocytosis [73, 75]. It has been suggested that ectopic expression of band 3 protein in sigmoidal colon adenocarcinoma cells is correlated with the development of secondary AIHA, causing anemia by enhancing phagocytosis of autoantibody-bound erythrocytes by macrophages [76].

### The mechanism of cancer treatment-induced anemia

In addition to cancer-induced anemia, cancer treatment-induced anemia is a common adverse event in chemotherapy, radiotherapy, targeted therapy, and immunotherapy (Table 1). Most types of chemotherapeutic agents result in anemia, the incidence and severity of which depends on the type of drug, dose, intensity, and number of cycles [77]. Myelosuppressive agents mainly trigger apoptosis of erythroid precursors via caspase activation [78]. Platinum-based chemotherapy has a direct toxic effect on erythropoiesis, and exhibits nephrotoxicity, which impairs EPO production [79]. Cisplatin-based agents increase ROS production, which downregulates EPO transcription, leading to reduced EPO synthesis in the kidneys [80]. Furthermore, cytostatic treatment with topotecan and cisplatin has been shown to trigger eryptosis, probably as a result of increased ceramide (a well-known eryptosis stimulator) on the erythrocyte surface [63, 81]. Common chemotherapy drugs, such as gemcitabine, mitomycin, and cisplatin, may also cause MAHA via dose-dependent toxicity or the development of drug-dependent antibodies [82]. Carboplatin and oxaliplatin-based chemotherapy may also induce AIHA [83, 84]. In addition to chemotherapeutic agents, radiotherapy may also lead to anemia by directly damaging the bone marrow or causing myelosuppression, thereby decreasing EPC production. Furthermore, chemoradiotherapy has gastrointestinal adverse events, such as nausea, vomiting, and diarrhea, which may decrease food intake and limit essential nutrients, such as iron and vitamins, for erythropoiesis [1].

**Table 1:** Etiology of cancer treatment-induced anemia

**Chemotherapy**
Suppression of erythropoiesis in bone marrow
Nephrotoxicity (impaired EPO production)
Increased eryptosis
Hemolysis (AIHA, MAHA)
Gastrointestinal reaction (nutritional deficiency)
Thrombocytopenia (blood loss)
**Radiotherapy**
Suppression of erythropoiesis in bone marrow
Gastrointestinal reaction (nutritional deficiency)
**Targeted therapy**
Suppression of erythropoiesis in bone marrow
Impaired iron metabolism
Increased eryptosis
**Immunotherapy**
ICIs- AIHA
CAR-T- CRS related anemia
**Surgery**
Blood loss
Nutritional deficiency (resection of stomach/bowel)

Anemia is also observed in patients receiving targeted therapy, with or without combined chemotherapy. Poly ADP-ribose polymerase (PARP) inhibitors, such as olaparib and niraparib, used to treat ovarian and breast cancer, may induce hematotoxicity by increasing replicative stress and decreasing erythroid precursors, as well as impairing iron metabolism [1, 85, 86]. Palbociclib, a cyclin-dependent kinase 4/6 inhibitor used for the treatment of metastatic breast cancer, causes dysplastic anemia by mimicking myelodysplastic syndrome [87]. Sunitinib, a tyrosine kinase inhibitor, is also associated with a high incidence of all-grade (50.4%) and high-grade anemia (6.2%) [88]. Gefitinib, an epidermal growth factor receptor-tyrosine kinase inhibitor (EGFR-TKI), can also induce erythrocyte shrinkage and cell membrane phospholipid scrambling [89], and combination of EGFR-TKI and chemotherapy is associated with an even higher incidence of all-grade anemia [90, 91]. B-cell lymphoma-2 (BCL-2)-inhibitor venetoclax monotherapy for relapsed or refractory multiple myeloma also shows a high incidence of anemia (23%) as a common grade III/IV event [92]. Hormone-related treatment, such as androgen deprivation therapy, also increases the risk of anemia in patients with prostate cancer [93]. Androgens can promote erythropoiesis by directly stimulating the incorporation of iron in EPCs and erythrocytes, and indirectly inhibiting hepcidin production via BMP/SMAD signaling, and upregulating renal EPO [93].

Although autologous anti-CD19 chimeric antigen receptor (CAR) T-cell therapy has shown an encouraging response in patients with refractory large B-cell lymphoma, it is accompanied by a high incidence of anemia (up to 43%) [94]. CAR T-cell therapy for patients with relapsed or refractory multiple myeloma has also exhibited toxic hematologic effects (approximately 45%) [95]. Furthermore, CAR T cell-induced cytokine release syndrome (CRS), mainly characterized by release of IL-1 and IL-6 from monocytes and macrophages, limits the broad applicability of this treatment [96, 97].

ICIs, such as anti-programmed cell death-1 (PD-1)/PD-L1 and anti-cytotoxic T lymphocyte-associated antigen-4 (CTLA-4), are another form of immunotherapy. Anemia, including all-grade (3.84%) and grade III or higher (0.74%) anemia, is the most common hematologic adverse event of PD-1 and PD-L1 inhibitors in advanced-stage cancer [98]. Moreover, ICIs can induce warm antibody AIHA by augmenting or redirecting immune surveillance [99], and the risk of AIHA is greater with PD-1 or PD-L1 monoclonal therapy than with CTLA-4-inhibitor treatment [100].

### Treatment of cancer-associated anemia

#### The current paradigm

Effective treatment of anemia during antineoplastic treatment could restore oxygenation, enhance therapy response, and reduce tumor invasion and metastasis [101]. Recently, cancer-associated anemia treatment has mainly focused on measures counteracting insufficient erythropoiesis, such as intravenous (IV) iron, erythropoietic stimulating agents (ESAs), and blood transfusion. However, retrospective clinical studies indicate that these treatments are inadequate [3].

For patients with cancer experiencing blood loss and AIDA, IV iron is the recommended therapy [3] as it is better tolerated than oral iron, and ESAs are less effective in treating AIDA and may exacerbate thrombocytosis [102].

The colonic microbiota of patients with colorectal cancer is different between IV iron and oral iron treatment. With IV iron treatment, the on- and off-tumor microbiota increased the abundance of enzymes involved in iron sequestration and anti-inflammatory or oncogenic metabolite production compared with oral iron treatment [103]. Furthermore, IV iron treatment can reduce ferritin expression in colorectal carcinoma and replenish iron stores more effectively than oral iron treatment [104]. In addition to being used as monotherapy, IV iron can reduce the frequency of blood transfusion and combined ESAs with IV iron treatment improves the efficacy of ESAs treatment and reduces the required ESAs dose [3, 105]. When combined with ESAs, IV iron is superior to oral iron for improving Hb level but neither of the iron combinations improve hematopoietic response [106, 107]. There is also no difference in the efficacy and safety between oral lactoferrin and IV iron, combined with recombinant human erythropoietin (rhEPO) therapy, for the treatment of chemotherapy-induced anemia in advanced cancer [108]. However, IV iron therapy has the risk of acute toxicity, including vasodilation, flushing, urticaria, and wheezing [3, 105].

Although treatment of cancer-associated anemia with ESAs increases Hb levels, which may modulate the efficacy of cancer radiotherapy by improving tumor oxygenation and reducing tumor HIF-1α expression [109, 110], and ESAs treatment prior to chemotherapy improves chemotherapeutic outcomes by mediating the prevention of anemia, tumor hypoxia, and increased drug delivery [111], the risks of ESAs have been widely reported. These risks include increased venous thromboembolism events, and tumor progression and recurrence via EPOR activation in tumor cells [112, 113]. In addition, rhEPO can induce angiogenesis by binding to EphB4, an alternative EPO receptor, instead of canonical EPOR [114]. Moreover, EPO inhibits chemotherapy-induced cell death in leukemia cells by increasing Mdm2-dependent p53 degradation and restoring anti-apoptotic Mcl-1 expression [115]. EPO also induces proliferation and protects against vincristine and etoposide in neuroblastoma cells via ERK1/2 and AKT activation [116]. Therefore, despite the efficacy of ESAs in the treatment of anemia, their ability to promote tumor progression limits the applicability of this treatment. Nowadays, ESAs are recommended carefully to patients with non-myeloid hematologic malignancies and iron-refractory anemia requiring frequent blood transfusions, or patients with decreased quality of life after the progressive stage of palliative myelosuppressive antineoplastic therapies. ESAs treatment is not suitable for patients with chemotherapy-associated anemia whose antineoplastic treatment is curative in intent, or for patients with non-chemotherapy-induced anemia, except low-risk myelodysplastic syndromes [3, 107]. Recently, novel distinctive carbon dots have been reported as a potential therapeutic agent for the treatment of cancer-associated anemia. Compared with EPO, carbon dots promote self-renewal of erythroid progenitors to increase Hb level and have no discernible effects on tumor proliferation and metastasis [117]. Thus, novel agents as alternatives for EPO are worth pursuing.

Blood transfusions in cancer patients have transient benefits and are associated with adverse effects, including thrombosis risk, transfusion-related acute lung injury, anaphylactic reactions, congestive heart failure, and iron overload [118]. Furthermore, numerous studies and meta-analyses have suggested that blood transfusions are associated with an increased risk of mortality and recurrence in patients with cancer during the perioperative period [24, 119]. Blood transfusions are therefore only recommended for the treatment of cancer-associated anemia grades II-IV when other therapies have failed [3].

#### The prospective paradigm

**Fig. 3:**
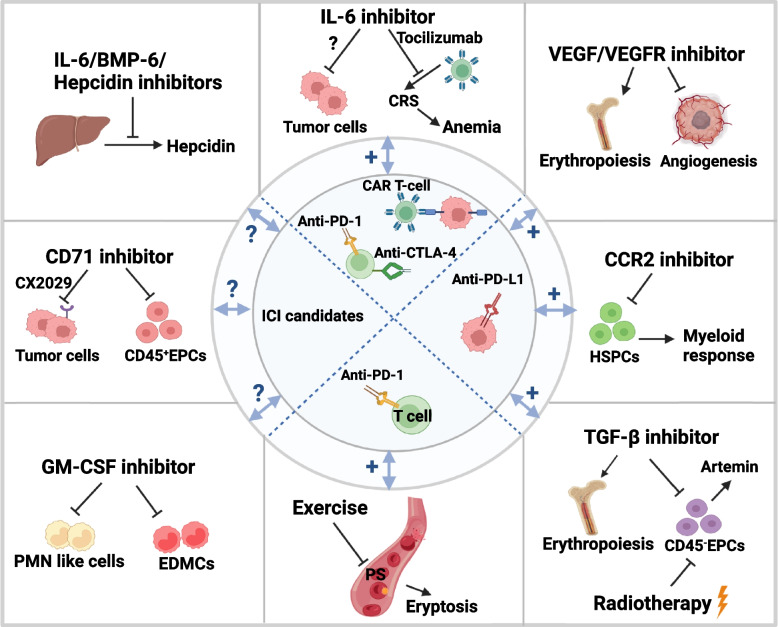
The prospective therapeutic strategies of cancer-associated anemia for synergistic immunotherapy. Combination the potential targets in cancer-associated anemia with immunotherapy could be the valuable therapeutic approach. Hepcidin inhibition with IL-6/BMP-6/hepcidin inhibitor improves anemia, and BMP-6/hepcidin inhibitors also have the potential to control tumor progression. Similarly, CD71 and CM-CSF inhibitor probably acting on EMH to curtail tumor growth, anemia and enhance immunotherapy efficacy. IL-6 inhibition not only reduces hepcidin production but also relives immunotherapy-related side effect, such as CAR-T induced CRS-related anemia, and synergistically with CAR T-cell therapy, anti-PD-1 and anti-CTLA-4 therapy. VEGF inhibition suppresses tumor angiogenesis and may improve erythropoiesis directly. CCR2 inhibitor relieves myeloid response in EMH. TGF-β inhibitor and radiotherapy decreases the accumulation of splenic CD45^−^EPCs, and TGF-β inhibitor may stimulate erythropoiesis directly. More importantly, VEGF/VEGFR inhibitor, CCR2 inhibitor and TGF-β inhibitor enhances anti-PD-L1 efficiency to alleviate tumor progression. Exercise can decrease eryptosis in tumor and boosts the efficacy of anti-PD-1 therapy

New strategies for the treatment of cancer-associated anemia are required and should, ideally, exhibit a synergistic anti-tumor effect. Combining potential cancer-induced anemia therapeutic targets, such as TGF-β, VEGF signaling, hepcidin, IL-6, CD71, CCL2/CCR2, GM-CSF, and exercise, with ICIs could therefore be an effective therapeutic approach (Fig. 3).

As mentioned previously, TGF-β inhibits erythropoiesis, blocking the maturation of megakaryocyte and erythroid progenitors in hepatocellular carcinoma-bearing hosts [16]. However, in multiple clinical trials, monotherapy with a TGF-β inhibitor showed a limited anti-tumor effect owing to its pleiotropic and dynamically controlled function and freely soluble ligand form [120]. TGF-β neutralization or inhibition does, however, downregulate splenic CD45^−^EPCs [16] and ameliorate cancer-induced anemia [17]. Because of its extensive expression and immunosuppressive function in most cancers, TGF-β has been combined with PD-L1 to develop anti-TGF-β/PD-L1 bispecific antibodies such as M7824 [121], SHR-1701 [122], and YM101 [123]. M7824 (bintrafusp alfa) treatment significantly increases Hb level in mouse models compared with the control, TGF-β trap, and anti-PD-L1 groups [124]. Furthermore, in a phase 1 trial of M7824, only one patient with an advanced solid tumor exhibited anemia (grade III, 5.3%) [121]. A phase 1 trial of SHR-1701 revealed promising anti-tumor activity against advanced solid tumors, and an acceptably low incidence of anemia (any grade, 15%) [125]. However, SHR-1701 treatment of recurrent or metastatic cervical cancer induced a greater anemia incidence (approximately 40%), which may, however, be attributed to prior platinum-based chemotherapy (87.5%) [122]. In addition to this reported phase 1 clinical trial results, other bispecific antibodies targeting TGF-β and PD-L1 are being investigated. Intriguingly, study has been demonstrated that both local tumor radiation and anti-PD-L1 treatment reduced CD45^−^EPCs via a remote effect to reduce artemin [126]. Based on this, combination radiation and ICIs may contribute to relieve cancer-associated anemia. It is noteworthy that both radiotherapy and immunotherapy could induce anemia, suggesting the importance of monitoring Hb level during treatment.

VEGF is a target for anti-angiogenesis and can induce anemia in tumor-bearing hosts. Phase 3 clinical trials of combined regimens of VEGF/VEGFR inhibitors and ICIs have shown significantly greater efficacy of combined therapy than that of VEGF/VEGFR-inhibitor monotherapy. Food and Drug Administration (FDA) has approved the combined atezolizumab and bevacizumab therapy for unresectable hepatocellular carcinoma [127]. In patients with advanced renal cell carcinoma, anemia was exhibited by 21% when treated with combined lenvatinib and pembrolizumab [128]. Combination therapy of biliary tract cancer with regorafenib and avelumab showed 15% anemia [129]. These clinical results suggest that targeting VEGF signaling and PD-L1 could lower the incidence of anemia.

The role of hepcidin in anemia and its upregulation in cancer cells suggests that it is a potential therapeutic target. Combination therapy with ESAs and short hairpin-RNA targeting hepcidin was demonstrated to reduce hepcidin production and alleviate anemia in a mouse model of inflammation-induced anemia [130]. Similarly, NOX-H94, a structured L-oligoribonucleotide targeting hepcidin, increased iron availability for erythropoiesis and inhibited decreased Hb levels in preclinical anemia of a chronic disease model [131]. Furthermore, LY2787106, a hepcidin monoclonal antibody, exhibited tolerance, safety, and a significant increase in serum iron levels in a phase 1 study of patients with cancer-associated anemia [132]. Lung cancer with high hepcidin expression is associated with decreased infiltration of immune cells and poor prognosis [133]. Colorectal cancer cells ectopically express hepcidin to accumulate iron, thereby promoting nucleotide synthesis and tumor cell proliferation [134]. However, blocking the hepcidin-FPN1 axis may increase iron availability not only for erythroid precursors, but also for cancer cells [135]. Therefore, further studies are needed to evaluate the benefit-risk ratio of hepcidin antagonists in cancer-associated anemia. Neutralizing antibodies of BMP-6 have been widely used to block hepcidin production in preclinical and clinical models of anemia in chronic diseases, such as chronic kidney disease, which also exhibit a potential role in reducing the need for EPO [136, 137]. However, multiple studies have suggested that the absence of or decreased BMP-6 is associated with poor survival and tumor progression in breast and non-small cell lung cancers [138, 139]. Furthermore, in prostate cancer, BMP-6 promotes tumor growth by inducing angiogenesis and castration resistance [140, 141]. Given this paradoxical role of BMP-6 in anemia and cancer, the efficacy of BMP-6 inhibition for the treatment of cancer-associated anemia requires further investigation.

Since IL-6 regulates circulating hepcidin levels under tumor conditions, blocking IL-6 with monoclonal antibodies tocilizumab or siltuximab may be suitable for the management of cancer-associated anemia. Additionally, IL-6 is involved in the survival and progression of tumor cells. IL-6/IL-6R inhibition may be an effective strategy to inhibit tumor progression and metastasis [142–144]. Palladium nanoplate-based IL-6R antagonists have been designed to deliver tocilizumab to the liver for hepcidin suppression, which alleviates cancer-associated anemia and simultaneously inhibits tumor progression, partly due to corrected anemia, in murine models [145]. Higher IL-6 levels and a greater number of T helper 17 (Th17) cells were observed in ICI-induced immune-related enterocolitis, and IL-6 inhibition increased the ICI-induced anti-tumor efficacy [146]. Combined ICIs and IL-6 inhibition may therefore be effective in reducing anemia, limiting immune-related toxicity and enhancing anti-tumor immunity [147]. Moreover, IL-6 is a representative cytokine in CAR-T cell-induced CRS, and the FDA has approved tocilizumab for the treatment of CRS after CAR T-cell therapy [148, 149]. However, another study showed that IL-6R inhibition might promote cholangiocarcinoma progression [150]. This suggests that the anti-tumor efficacy of combined IL-6/IL-6R inhibition and ICIs for cancer-associated anemia may be tumor-dependent.

Tumor-induced extramedullary hematopoiesis produces abundant CD71 (transferrin receptor 1) positive EPCs suggesting that neutralizing CD71 could be an effective strategy to deplete these robust immunosuppressors. Using an anti-CD71 antibody to deplete CD45^+^EPCs in tumor-bearing mice relieved CD8^+^T cell suppression and ameliorated cancer-induced anemia [56]. Furthermore, CD71 has been designed as a probody-drug (CX-2029) for the treatment of advanced solid tumors. Unfortunately, 67% of patients receiving CX-2029 treatment exhibited anemia, which is greater than the incidence of cancer-induced anemia (40%) [151]. Although CX-2029 exhibited high anti-tumor efficacy in patient-derived xenograft mouse models, as well as safety in cynomolgus monkeys [152], this anti-tumor effect seems to directly target tumor cells but not splenic EPCs because of the masked form of CX-2029 in normal cells. Further studies regarding the applicability of combined CX-2029 and ICI therapy are required.

In addition to the depletion of EPCs in extramedullary organs, repression of EPC migration or differentiation could be another potential therapeutic strategy. CCR2 knockout reduces splenic recruitment of circulating HSPCs in hepatoma mice and synergistically enhances anti-PD-L1 efficacy [59]. Moreover, PF-04136309 is a CCR2 inhibitor that targets tumor-associated macrophages, and a phase 1b trial revealed that PF-04136309 in combination with FOLFIRINOX chemotherapy decreases the incidence of grade III anemia (8%) in pancreatic cancer compared with FOLFIRINOX alone [153]. In addition, antibody-mediated GM-CSF neutralization decreases EDMCs in tumors [11]. Although erythroid-myeloid differentiation can be blocked with anti-GM-CSF, the oncolytic virus armed with GM-CSF was approved by the FDA to treat melanoma through the induction of specific anti-tumor immunity [154]. Targeting GM-CSF could therefore reduce the accumulation of immunosuppressive myeloid cells as well as anti-tumor dendritic cells.

Preclinical tumor models have indicated that exercise might ameliorate cancer-associated anemia by reducing circulating blood lactate and IL-1β levels to promote erythrocyte survival [65]. Furthermore, aerobic exercise promotes anti-tumor immunity and reduces tumor growth in pancreatic cancer through the accumulation of tumor-infiltrating IL15Rα^+^ CD8 T cells. Additionally, exercise boosts the sensitivity of pancreatic tumors to chemotherapy and anti-PD-1 therapy [155]. Targeting cancer-induced anemia with aerobic exercise may therefore synergistically enhance ICI treatment in patients with cancer.

In addition, effective antineoplastic therapy in patients with cancer could induce regression of the tumor mass to reduce tumor factors for anemia, thereby gaining adequate nutritional support and attenuating cancer-associated anemia.

## Conclusion

Anemia is a common occurrence in patients with cancer, with or without antineoplastic therapy, and is associated with significantly decreased quality of life and may have a negative impact on prognosis. Moreover, cancer-associated anemia is a risk factor for ICIs efficacy [11]. Therefore, targeting cancer-associated anemia contributes to synergistic immunotherapy. Some currently available drugs exhibit efficacy in cancer-associated anemia treatments, as well as anti-tumor effects, such as relieving the immunosuppressive tumor microenvironment, anti-angiogenesis, and normalizing metabolism. Combining these multitarget drugs with immunotherapy could achieve a triple-win effect. Moreover, some of these drugs have been approved for cancer treatment, even if they do not ameliorate anemia. Future clinical trials should therefore evaluate these prospective combination strategies for the synergistic treatment of cancer and cancer-induced anemia.

## Data Availability

No applicable.

## Abbreviations

AIDA: Absolute iron deficiency anemia
AIHA: Autoimmune hemolytic anemia
Ang II: Angiotensin II
BCL-2: B-cell lymphoma-2
BFU-E: Burst-forming unit-erythroid
BMP: Bone morphogenetic protein
CAR: Anti-CD19 chimeric antigen receptor
CFU-E: Colony-forming unit-erythroid
CKD: Chronic kidney disease
CRS: Cytokine release syndrome
CTCAE: Common Terminology Criteria for Adverse Events (CTCAE)
CTLA-4: Cytotoxic T lymphocyte-associated antigen-4
DMT1: Divalent metal transporter 1
EDMCs: Erythroid differentiated myeloid cells
EGFR-TKI: Epidermal growth factor receptor-tyrosine kinase inhibitor
EMH: Extramedullary hematopoiesis
EPC: Erythroid progenitor cell
EPO: Erythropoietin
EPOR: EPO receptor
Hb: Hemoglobin
ESAs: Erythropoietic stimulating agents
FDA: Food and Drug Administration
FIDA: Functional iron deficiency anemia
FPN: Ferroportin
GDF-15: Growth differentiation factor-15
GM-CSF: Granulocyte–macrophage colony-stimulating factor
G-CSF: Granulocyte-CSF
HIF-1α: hypoxia inducible factor-1α
HSC: Hematopoietic stem cell
ICIs: Immune checkpoint inhibitors
IL: Interleukin
IL-6R: IL-6 receptor
IV: Intravenous
MAHA: Microangiopathic hemolytic anemia
PARP: Poly ADP-ribose polymerase
PDGF: Platelet-derived growth factor
PD-1: Programmed cell death-1
PD-L1: Programmed cell death ligand-1
PMN: Polymorphonuclear
PS: Phosphatidylserine
rhEPO: Recombinant human erythropoietin
ROS: Reactive oxygen species
RPMs: Red pulp macrophages
SMAD: Small-mothers-against-decapentaplegic
STAT3: Signal transducer and activator of transcription 3
TAMs: Tumor associated macrophages
TGF-β: Transforming growth factor-β
Th17: T helper 17
TLR: Toll-like receptor
TMB: Tumor mutational burden
WHO: World Health Organization
VEGF: Vascular endothelial growth factor

## References

[1] Madeddu C, Neri M, Sanna E, Oppi S, Maccio A (2021). Experimental drugs for chemotherapy- and cancer-related anemia. J Exp Pharmacol.

[2] Spivak JL (2005). The anaemia of cancer: Death by a thousand cuts. Nat Rev Cancer.

[3] Gilreath JA, Rodgers GM (2020). How I treat cancer-associated anemia. Blood.

[4] Chen C, Song Z, Wang W, Zhou J (2021). Baseline anemia and anemia grade are independent prognostic factors for stage IV non-small cell lung cancer. Mol Clin Oncol.

[5] Ludwig H, Van Belle S, Barrett-Lee P, Birgegard G, Bokemeyer C, Gascon P (2004). The European Cancer Anaemia Survey (ECAS): a large, multinational, prospective survey defining the prevalence, incidence, and treatment of anaemia in cancer patients. Eur J Cancer.

[6] Sharma P, Siddiqui BA, Anandhan S, Yadav SS, Subudhi SK, Gao J (2021). The next decade of immune checkpoint therapy. Cancer Discov.

[7] Topalian SL, Hodi FS, Brahmer JR, Gettinger SN, Smith DC, McDermott DF (2012). Safety, activity, and immune correlates of anti-PD-1 antibody in cancer. N Engl J Med.

[8] Marabelle A, Fakih M, Lopez J, Shah M, Shapira-Frommer R, Nakagawa K (2020). Association of tumour mutational burden with outcomes in patients with advanced solid tumours treated with pembrolizumab: prospective biomarker analysis of the multicohort, open-label, phase 2 KEYNOTE-158 study. Lancet Oncol.

[9] Le DT, Uram JN, Wang H, Bartlett BR, Kemberling H, Eyring AD (2015). PD-1 Blockade in tumors with mismatch-repair deficiency. N Engl J Med.

[10] Sautes-Fridman C, Petitprez F, Calderaro J, Fridman WH (2019). Tertiary lymphoid structures in the era of cancer immunotherapy. Nat Rev Cancer.

[11] Long H, Jia Q, Wang L, Fang W, Wang Z, Jiang T (2022). Tumor-induced erythroid precursor-differentiated myeloid cells mediate immunosuppression and curtail anti-PD-1/PD-L1 treatment efficacy. Cancer Cell.

[12] Mandula JK, Rodriguez PC (2022). Tumor-directed dysregulation of erythroid progenitors drives immunosuppressive myeloid cells. Cancer Cell.

[13] Blank U, Karlsson S (2015). TGF-beta signaling in the control of hematopoietic stem cells. Blood.

[14] Zermati Y, Fichelson S, Valensi F, Freyssinier JM, Rouyer-Fessard P, Cramer E (2000). Transforming growth factor inhibits erythropoiesis by blocking proliferation and accelerating differentiation of erythroid progenitors. Exp Hematol.

[15] Matsuzaki K, Date M, Furukawa F, Tahashi Y, Matsushita M, Sakitani K (2000). Autocrine stimulatory mechanism by transforming growth factor beta in human hepatocellular carcinoma. Cancer Res.

[16] Han Y, Liu Q, Hou J, Gu Y, Zhang Y, Chen Z (2018). Tumor-induced generation of splenic erythroblast-like Ter-cells promotes tumor progression. Cell.

[17] Wang BY, Wang Y, Chen HN, Yao SY, Lai XF, Qiu Y (2021). Inhibition of TGF beta improves hematopoietic stem cell niche and ameliorates cancer-related anemia. Stem Cell Res Ther.

[18] Feng CC, Ding GX, Song NH, Li X, Wu Z, Jiang HW (2013). Paraneoplastic hormones: parathyroid hormone-related protein (PTHrP) and erythropoietin (EPO) are related to vascular endothelial growth factor (VEGF) expression in clear cell renal cell carcinoma. Tumour Biol.

[19] Jubb AM, Pham TQ, Hanby AM, Frantz GD, Peale FV, Wu TD (2004). Expression of vascular endothelial growth factor, hypoxia inducible factor 1alpha, and carbonic anhydrase IX in human tumours. J Clin Pathol.

[20] Greenwald AC, Licht T, Kumar S, Oladipupo SS, Iyer S, Grunewald M (2019). VEGF expands erythropoiesis via hypoxia-independent induction of erythropoietin in noncanonical perivascular stromal cells. J Exp Med.

[21] Lim S, Zhang Y, Zhang D, Chen F, Hosaka K, Feng N (2014). VEGFR2-mediated vascular dilation as a mechanism of VEGF-induced anemia and bone marrow cell mobilization. Cell Rep.

[22] Xue Y, Religa P, Cao R, Hansen AJ, Lucchini F, Jones B (2008). Anti-VEGF agents confer survival advantages to tumor-bearing mice by improving cancer-associated systemic syndrome. Proc Natl Acad Sci U S A.

[23] Ganz T, Nemeth E (2016). Iron balance and the role of hepcidin in chronic kidney disease. Semin Nephrol.

[24] Busti F, Marchi G, Ugolini S, Castagna A, Girelli D (2018). Anemia and iron deficiency in cancer patients: role of iron replacement therapy. Pharmaceuticals (Basel).

[25] Weiss G, Goodnough LT (2005). Anemia of chronic disease. N Engl J Med.

[26] Gilreath JA, Stenehjem DD, Rodgers GM (2012). Total dose iron dextran infusion in cancer patients: is it SaFe2+?. J Natl Compr Canc Netw.

[27] Theurl I, Theurl M, Seifert M, Mair S, Nairz M, Rumpold H (2008). Autocrine formation of hepcidin induces iron retention in human monocytes. Blood.

[28] Wessling-Resnick M (2010). Iron homeostasis and the inflammatory response. Annu Rev Nutr.

[29] Fraenkel PG (2017). Anemia of inflammation: a review. Med Clin North Am.

[30] Ganz T (2011). Hepcidin and iron regulation, 10 years later. Blood.

[31] Zhang AS, Enns CA. Molecular mechanisms of normal iron homeostasis. Hematology Am Soc Hematol Educ Program 2009;1:207–14. 10.1182/asheducation-2009.1.207 PMC5831338 20008200

[32] Henry DH, Dahl NV, Auerbach M, Tchekmedyian S, Laufman LR (2007). Intravenous ferric gluconate significantly improves response to epoetin alfa versus oral iron or no iron in anemic patients with cancer receiving chemotherapy. Oncologist.

[33] Rivera S, Liu L, Nemeth E, Gabayan V, Sorensen OE, Ganz T (2005). Hepcidin excess induces the sequestration of iron and exacerbates tumor-associated anemia. Blood.

[34] Falkensammer CE, Thurnher M, Leonhartsberger N, Ramoner R (2011). C-reactive protein is a strong predictor for anaemia in renal cell carcinoma: role of IL-6 in overall survival. BJU Int.

[35] Madeddu C, Gramignano G, Kotsonis P, Coghe F, Atzeni V, Scartozzi M (2018). Microenvironmental M1 tumor-associated macrophage polarization influences cancer-related anemia in advanced ovarian cancer: key role of interleukin-6. Haematologica.

[36] Nieken J, Mulder NH, Buter J, Vellenga E, Limburg PC, Piers DA (1995). Recombinant human interleukin-6 induces a rapid and reversible anemia in cancer patients. Blood.

[37] Mori K, Fujimoto-Ouchi K, Onuma E, Noguchi M, Shimonaka Y, Yasuno H (2009). Novel models of cancer-related anemia in mice inoculated with IL-6-producing tumor cells. Biomed Res.

[38] Nemeth E, Rivera S, Gabayan V, Keller C, Taudorf S, Pedersen BK (2004). IL-6 mediates hypoferremia of inflammation by inducing the synthesis of the iron regulatory hormone hepcidin. J Clin Invest.

[39] Atkins MB, Kappler K, Mier JW, Isaacs RE, Berkman EM (1995). Interleukin-6-associated anemia: determination of the underlying mechanism. Blood.

[40] Maes K, Nemeth E, Roodman GD, Huston A, Esteve F, Freytes C (2010). In anemia of multiple myeloma, hepcidin is induced by increased bone morphogenetic protein 2. Blood.

[41] Cheng Z, Yan M, Lu Y, Pan XT (2020). Expression of serum BMP6 and hepcidin in cancer-related anemia. Hematology.

[42] Andriopoulos B, Corradini E, Xia Y, Faasse SA, Chen SZ, Grgurevic L (2009). BMP6 is a key endogenous regulator of hepcidin expression and iron metabolism. Nat Genet.

[43] Jiang F, Yu WJ, Wang XH, Tang YT, Guo L, Jiao XY (2014). Regulation of hepcidin through GDF-15 in cancer-related anemia. Clin Chim Acta.

[44] Richmond TD, Chohan M, Barber DL (2005). Turning cells red: signal transduction mediated by erythropoietin. Trends Cell Biol.

[45] Zhao W, Kitidis C, Fleming MD, Lodish HF, Ghaffari S (2006). Erythropoietin stimulates phosphorylation and activation of GATA-1 via the PI3-kinase/AKT signaling pathway. Blood.

[46] Angelillo-Scherrer A, Burnier L, Lambrechts D, Fish RJ, Tjwa M, Plaisance S (2008). Role of Gas6 in erythropoiesis and anemia in mice. J Clin Invest.

[47] Ozguroglu M, Arun B, Demir G, Demirelli F, Mandel NM, Buyukunal E (2000). Serum erythropoietin level in anemic cancer patients. Med Oncol.

[48] Miller CB, Jones RJ, Piantadosi S, Abeloff MD, Spivak JL (1990). Decreased erythropoietin response in patients with the anemia of cancer. N Engl J Med.

[49] Gilreath JA, Stenehjem DD, Rodgers GM (2014). Diagnosis and treatment of cancer-related anemia. Am J Hematol.

[50] Kling PJ, Dragsten PR, Roberts RA, Dos Santos B, Brooks DJ, Hedlund BE (1996). Iron deprivation increases erythropoietin production in vitro, in normal subjects and patients with malignancy. Br J Haematol.

[51] Yang X, Chen D, Long H, Zhu B (2020). The mechanisms of pathological extramedullary hematopoiesis in diseases. Cell Mol Life Sci.

[52] Vignjevic Petrinovic S, Jaukovic A, Milosevic M, Bugarski D, Budec M (2022). Targeting stress erythropoiesis pathways in cancer. Front Physiol.

[53] Xue Y, Chen F, Zhang D, Lim S, Cao Y (2009). Tumor-derived VEGF modulates hematopoiesis. J Angiogenes Res.

[54] Xue Y, Lim S, Yang Y, Wang Z, Jensen LD, Hedlund EM (2011). PDGF-BB modulates hematopoiesis and tumor angiogenesis by inducing erythropoietin production in stromal cells. Nat Med.

[55] Liu M, Jin X, He X, Pan L, Zhang X, Zhao Y (2015). Macrophages support splenic erythropoiesis in 4T1 tumor-bearing mice. PLoS ONE.

[56] Zhao L, He R, Long H, Guo B, Jia Q, Qin D (2018). Late-stage tumors induce anemia and immunosuppressive extramedullary erythroid progenitor cells. Nat Med.

[57] Kamran N, Li Y, Sierra M, Alghamri MS, Kadiyala P, Appelman HD (2018). Melanoma induced immunosuppression is mediated by hematopoietic dysregulation. Oncoimmunology.

[58] Cortez-Retamozo V, Etzrodt M, Newton A, Ryan R, Pucci F, Sio SW (2013). Angiotensin II drives the production of tumor-promoting macrophages. Immunity.

[59] Wu C, Ning H, Liu M, Lin J, Luo S, Zhu W (2018). Spleen mediates a distinct hematopoietic progenitor response supporting tumor-promoting myelopoiesis. J Clin Invest.

[60] DuPre SA, Hunter KW (2007). Murine mammary carcinoma 4T1 induces a leukemoid reaction with splenomegaly: association with tumor-derived growth factors. Exp Mol Pathol.

[61] Chen J, Qiao YD, Li X, Xu JL, Ye QJ, Jiang N (2021). Intratumoral CD45(+)CD71(+) erythroid cells induce immune tolerance and predict tumor recurrence in hepatocellular carcinoma. Cancer Lett.

[62] Lang E, Qadri SM, Lang F (2012). Killing me softly - suicidal erythrocyte death. Int J Biochem Cell Biol.

[63] Bissinger R, Schumacher C, Qadri SM, Honisch S, Malik A, Gotz F (2016). Enhanced eryptosis contributes to anemia in lung cancer patients. Oncotarget.

[64] Lang E, Bissinger R, Qadri SM, Lang F (2017). Suicidal death of erythrocytes in cancer and its chemotherapy: A potential target in the treatment of tumor-associated anemia. Int J Cancer.

[65] Furrer R, Jauch AJ, Rao TN, Dilbaz S, Rhein P, Steurer SA (2021). Remodeling of metabolism and inflammation by exercise ameliorates tumor-associated anemia. Sci Adv.

[66] Olszewska M, Wiatrow J, Bober J, Stachowska E, Golembiewska E, Jakubowska K (2012). Oxidative stress modulates the organization of erythrocyte membrane cytoskeleton. Postepy Hig Med Dosw (Online).

[67] Lechner K, Obermeier HL (2012). Cancer-related microangiopathic hemolytic anemia: clinical and laboratory features in 168 reported cases. Medicine (Baltimore).

[68] Thomas MR, Scully M (2021). How I treat microangiopathic hemolytic anemia in patients with cancer. Blood.

[69] Johnson RA, Roodman GD (1989). Hematologic manifestations of malignancy. Dis Mon.

[70] Steinberg D (1989). Anemia and cancer. CA Cancer J Clin.

[71] Werner TL, Agarwal N, Carney HM, Rodgers GM (2007). Management of cancer-associated thrombotic microangiopathy: what is the right approach?. Am J Hematol.

[72] von Bubnoff N, Sandherr M, Schneller F, Peschel C (2000). Thrombotic thrombocytopenic purpura in metastatic carcinoma of the breast. Am J Clin Oncol.

[73] Gaspar BL, Sharma P, Das R (2015). Anemia in malignancies: pathogenetic and diagnostic considerations. Hematology.

[74] Puthenparambil J, Lechner K, Kornek G (2010). Autoimmune hemolytic anemia as a paraneoplastic phenomenon in solid tumors: A critical analysis of 52 cases reported in the literature. Wien Klin Wochenschr.

[75] Morris PG, Swords R, Sukor S, Fortune A, O'Donnell DM, Conneally E (2008). Autoimmune hemolytic anemia associated with ovarian cancer. J Clin Oncol.

[76] Kawamoto S, Kamesaki T, Masutani R, Kitao A, Hatanaka K, Imakita M (2019). Ectopic expression of band 3 anion transport protein in colorectal cancer revealed in an autoimmune hemolytic anemia patient. Hum Pathol.

[77] Cheng K, Zhao F, Gao F, Dong H, Men HT, Chen Y (2012). Factors potentially associated with chemotherapy-induced anemia in patients with solid cancers. Asian Pac J Cancer Prev.

[78] Zeuner A, Pedini F, Signore M, Testa U, Pelosi E, Peschle C (2003). Stem cell factor protects erythroid precursor cells from chemotherapeutic agents via up-regulation of BCL-2 family proteins. Blood.

[79] Perazella MA, Moeckel GW (2010). Nephrotoxicity from chemotherapeutic agents: clinical manifestations, pathobiology, and prevention/therapy. Semin Nephrol.

[80] Maccio A, Madeddu C (2013). Cisplatin : an old drug with a newfound efficacy – from mechanisms of action to cytotoxicity. Expert Opin Pharmacother.

[81] Lang F, Gulbins E, Lang PA, Zappulla D, Foller M (2010). Ceramide in suicidal death of erythrocytes. Cell Physiol Biochem.

[82] Morton JM, George JN (2016). Microangiopathic hemolytic anemia and thrombocytopenia in patients with cancer. J Oncol Pract.

[83] Ogura T, Tajika M, Niwa Y, Kawai H, KondoSawaki SA (2011). [Recurrent autoimmune hemolytic anemia induced by XELOX chemotherapy for colon cancer]. Nihon Shokakibyo Gakkai Zasshi.

[84] Haley KM, Russell TB, Boshkov L, Leger RM, Garratty G, Recht M (2014). Fatal carboplatin-induced immune hemolytic anemia in a child with a brain tumor. J Blood Med.

[85] Farres J, Llacuna L, Martin-Caballero J, Martinez C, Lozano JJ, Ampurdanes C (2015). PARP-2 sustains erythropoiesis in mice by limiting replicative stress in erythroid progenitors. Cell Death Differ.

[86] Pelham C, Jimenez T, Rodova M, Rudolph A, Chipps E, Islam MR (2013). Regulation of HFE expression by poly(ADP-ribose) polymerase-1 (PARP1) through an inverted repeat DNA sequence in the distal promoter. Biochim Biophys Acta.

[87] Anampa J, Haque T, Murakhovskaya I, Wang Y, Bachiashvili K, Papazoglu C (2018). Macrocytosis and dysplastic anemia is associated with the cyclin-dependent kinase 4/6 inhibitor palbociclib in metastatic breast cancer. Haematologica.

[88] Funakoshi T, Latif A, Galsky MD (2013). Risk of hematologic toxicities in cancer patients treated with sunitinib: a systematic review and meta-analysis. Cancer Treat Rev.

[89] AlMamunBhuyan A, Wagner T, Cao H, Lang F (2017). Triggering of suicidal erythrocyte death by Gefitinib. Cell Physiol Biochem.

[90] Wu Q, Luo W, Li W, Wang T, Huang L, Xu F (2021). First-generation EGFR-TKI plus chemotherapy versus EGFR-TKI alone as first-line treatment in advanced NSCLC With EGFR activating mutation: a systematic review and meta-analysis of randomized controlled trials. Front Oncol.

[91] Cheng Y, Murakami H, Yang PC, He J, Nakagawa K, Kang JH (2016). Randomized phase II trial of Gefitinib with and without pemetrexed as first-line therapy in patients with advanced nonsquamous non-small-cell lung cancer with activating epidermal growth factor receptor mutations. J Clin Oncol.

[92] Kumar S, Kaufman JL, Gasparetto C, Mikhael J, Vij R, Pegourie B (2017). Efficacy of venetoclax as targeted therapy for relapsed/refractory t(11;14) multiple myeloma. Blood.

[93] Hicks BM, Klil-Drori AJ, Yin H, Campeau L, Azoulay L (2017). Androgen deprivation therapy and the risk of anemia in men with prostate cancer. Epidemiology.

[94] Neelapu SS, Locke FL, Bartlett NL, Lekakis LJ, Miklos DB, Jacobson CA (2017). Axicabtagene ciloleucel CAR T-Cell Therapy in refractory large b-cell lymphoma. N Engl J Med.

[95] Raje N, Berdeja J, Lin Y, Siegel D, Jagannath S, Madduri D (2019). Anti-BCMA CAR T-Cell Therapy bb2121 in relapsed or refractory multiple myeloma. N Engl J Med.

[96] Norelli M, Camisa B, Barbiera G, Falcone L, Purevdorj A, Genua M (2018). Monocyte-derived IL-1 and IL-6 are differentially required for cytokine-release syndrome and neurotoxicity due to CAR T cells. Nat Med.

[97] Giavridis T, van der Stegen SJC, Eyquem J, Hamieh M, Piersigilli A, Sadelain M (2018). CAR T cell-induced cytokine release syndrome is mediated by macrophages and abated by IL-1 blockade. Nat Med.

[98] Wang Y, Zhou S, Yang F, Qi X, Wang X, Guan X (2019). Treatment-related adverse events of PD-1 and PD-L1 inhibitors in clinical trials: a systematic review and meta-analysis. JAMA Oncol.

[99] Tanios GE, Doley PB, Munker R (2019). Autoimmune hemolytic anemia associated with the use of immune checkpoint inhibitors for cancer: 68 cases from the food and drug administration database and review. Eur J Haematol.

[100] Barcellini W, Zaninoni A, Giannotta JA, Fattizzo B (2020). New insights in autoimmune hemolytic anemia: from pathogenesis to therapy stage 1. J Clin Med.

[101] Koukourakis MI, Giatromanolaki A, Polychronidis A, Simopoulos C, Gatter KC, Harris AL (2006). Endogenous markers of hypoxia/anaerobic metabolism and anemia in primary colorectal cancer. Cancer Sci.

[102] Henry DH, Dahl NV, Auerbach MA (2012). Thrombocytosis and venous thromboembolism in cancer patients with chemotherapy induced anemia may be related to ESA induced iron restricted erythropoiesis and reversed by administration of IV iron. Am J Hematol.

[103] Phipps O, Al-Hassi HO, Quraishi MN, Dickson EA, Segal J, Steed H (2021). Oral and intravenous iron therapy differentially alter the on- and off-tumor microbiota in anemic colorectal cancer patients. Cancers (Basel).

[104] Al-Hassi HO, Ng O, Evstatiev R, Mangalika M, Worton N, Jambrich M (2021). Intravenous iron is non-inferior to oral iron regarding cell growth and iron metabolism in colorectal cancer associated with iron-deficiency anaemia. Sci Rep.

[105] Rampton D, Folkersen J, Fishbane S, Hedenus M, Howaldt S, Locatelli F (2014). Hypersensitivity reactions to intravenous iron: guidance for risk minimization and management. Haematologica.

[106] Mhaskar R, Wao H, Miladinovic B, Kumar A, Djulbegovic B (2016). The role of iron in the management of chemotherapy-induced anemia in cancer patients receiving erythropoiesis-stimulating agents. Cochrane Database Syst Rev.

[107] Bohlius J, Bohlke K, Castelli R, Djulbegovic B, Lustberg MB, Martino M (2019). Management of cancer-associated anemia with erythropoiesis-stimulating agents: ASCO/ASH clinical practice guideline update. J Clin Oncol.

[108] Maccio A, Madeddu C, Gramignano G, Mulas C, Sanna E, Mantovani G (2010). Efficacy and safety of oral lactoferrin supplementation in combination with rHuEPO-beta for the treatment of anemia in advanced cancer patients undergoing chemotherapy: open-label, randomized controlled study. Oncologist.

[109] Lovey J, Bereczky B, Gilly R, Kenessey I, Raso E, Simon E (2008). Recombinant human erythropoietin alpha improves the efficacy of radiotherapy of a human tumor xenograft, affecting tumor cells and microvessels. Strahlenther Onkol.

[110] Kelleher DK, Mattheinsen U, Thews O, Vaupel P (1996). Blood flow, oxygenation, and bioenergetic status of tumors after erythropoietin treatment in normal and anemic rats. Cancer Res.

[111] Shannon AM, Bouchier-Hayes DJ, Condron CM, Toomey D (2005). Correction of anaemia through the use of darbepoetin alfa improves chemotherapeutic outcome in a murine model of Lewis lung carcinoma. Br J Cancer.

[112] Bennett CL, Silver SM, Djulbegovic B, Samaras AT, Blau CA, Gleason KJ (2008). Venous thromboembolism and mortality associated with recombinant erythropoietin and darbepoetin administration for the treatment of cancer-associated anemia. JAMA.

[113] Aapro M, Jelkmann W, Constantinescu SN, Leyland-Jones B (2012). Effects of erythropoietin receptors and erythropoiesis-stimulating agents on disease progression in cancer. Br J Cancer.

[114] Pradeep S, Huang J, Mora EM, Nick AM, Cho MS, Wu SY (2015). Erythropoietin stimulates tumor growth via EphB4. Cancer Cell.

[115] Pham TD, Ma W, Miller D, Kazakova L, Benchimol S (2019). Erythropoietin inhibits chemotherapy-induced cell death and promotes a senescence-like state in leukemia cells. Cell Death Dis.

[116] Vazquez-Mellado MJ, Aguilar C, Rocha-Zavaleta L (2015). Erythropoietin protects neuroblastoma cells against etoposide and vincristine by activating ERK and AKT pathways but has no effect in kidney cells. Life Sci.

[117] Xu Y, Wang B, Zhang M, Zhang J, Li Y, Jia P (2022). Carbon dots as a potential therapeutic agent for the treatment of cancer-related anemia. Adv Mater.

[118] Palomero-Rodriguez MA, Laporta-Baez Y, Sanchez-Conde MP, Mollinedo F (2014). Inflammatory response, immunosuppression, and cancer recurrence after perioperative blood transfusion. Br J Anaesth.

[119] Sun C, Wang Y, Yao HS, Hu ZQ (2015). Allogeneic blood transfusion and the prognosis of gastric cancer patients: systematic review and meta-analysis. Int J Surg.

[120] Teixeira AF, Ten Dijke P, Zhu HJ (2020). On-target anti-TGF-beta therapies are not succeeding in clinical cancer treatments: what are remaining challenges?. Front Cell Dev Biol.

[121] Strauss J, Heery CR, Schlom J, Madan RA, Cao L, Kang Z (2018). Phase I trial of M7824 (MSB0011359C), a bifunctional fusion protein targeting PD-L1 and TGFbeta, in advanced solid tumors. Clin Cancer Res.

[122] Feng J, Tang D, Wang J, Zhou Q, Peng J, Lou H (2022). SHR-1701, a bifunctional fusion protein targeting PD-L1 and TGF-beta, for recurrent or metastatic cervical cancer: a clinical expansion cohort of phase 1 study. Clin Cancer Res.

[123] Yi M, Zhang J, Li A, Niu M, Yan Y, Jiao Y (2021). The construction, expression, and enhanced anti-tumor activity of YM101: a bispecific antibody simultaneously targeting TGF-beta and PD-L1. J Hematol Oncol.

[124] Lan Y, Moustafa M, Knoll M, Xu C, Furkel J, Lazorchak A (2021). Simultaneous targeting of TGF-beta/PD-L1 synergizes with radiotherapy by reprogramming the tumor microenvironment to overcome immune evasion. Cancer Cell.

[125] Liu D, Zhou J, Wang Y, Li M, Jiang H, Liu Y (2022). Bifunctional anti-PD-L1/TGF-betaRII agent SHR-1701 in advanced solid tumors: a dose-escalation, dose-expansion, and clinical-expansion phase 1 trial. BMC Med.

[126] Hou Y, Liang HL, Yu X, Liu Z, Cao X, Rao E (2021). Radiotherapy and immunotherapy converge on elimination of tumor-promoting erythroid progenitor cells through adaptive immunity. Sci Transl Med.

[127] Bejjani AC, Finn RS (2022). Hepatocellular carcinoma: pick the winner-tyrosine kinase inhibitor versus immuno-oncology agent-based combinations. J Clin Oncol.

[128] Finn RS, Ikeda M, Zhu AX, Sung MW, Baron AD, Kudo M (2020). Phase Ib study of Lenvatinib plus Pembrolizumab in patients with unresectable hepatocellular carcinoma. J Clin Oncol.

[129] Cousin S, Cantarel C, Guegan JP, Mazard T, Gomez-Roca C, Metges JP (2022). Regorafenib-avelumab combination in patients with biliary tract cancer (REGOMUNE): a single-arm, open-label, phase II trial. Eur J Cancer.

[130] Sasu BJ, Cooke KS, Arvedson TL, Plewa C, Ellison AR, Sheng J (2010). Antihepcidin antibody treatment modulates iron metabolism and is effective in a mouse model of inflammation-induced anemia. Blood.

[131] Schwoebel F, van Eijk LT, Zboralski D, Sell S, Buchner K, Maasch C (2013). The effects of the anti-hepcidin Spiegelmer NOX-H94 on inflammation-induced anemia in cynomolgus monkeys. Blood.

[132] Vadhan-Raj S, Abonour R, Goldman JW, Smith DA, Slapak CA, Ilaria RL (2017). A first-in-human phase 1 study of a hepcidin monoclonal antibody, LY2787106, in cancer-associated anemia. J Hematol Oncol.

[133] Fan Y, Liu B, Chen F, Song Z, Han B, Meng Y (2021). Hepcidin upregulation in lung cancer: a potential therapeutic target associated with immune infiltration. Front Immunol.

[134] Schwartz AJ, Goyert JW, Solanki S, et al. Hepcidin sequesters iron to sustain nucleotide metabolism and mitochondrial function in colorectal cancer epithelial cells. Nat Metab. 2021;3(7):969–82. 10.1038/s42255-021-00406-7 PMC8316354 34155415

[135] Weiler S, Nairz M (2021). TAM-ing the CIA-tumor-associated macrophages and their potential role in unintended side effects of therapeutics for cancer-induced anemia. Front Oncol.

[136] Sheetz M, Barrington P, Callies S, Berg PH, McColm J, Marbury T (2019). Targeting the hepcidin-ferroportin pathway in anaemia of chronic kidney disease. Br J Clin Pharmacol.

[137] Petzer V, Tymoszuk P, Asshoff M, Carvalho J, Papworth J, Deantonio C (2020). A fully human anti-BMP6 antibody reduces the need for erythropoietin in rodent models of the anemia of chronic disease. Blood.

[138] Xiong W, Wang L, Yu F (2019). Expression of bone morphogenetic protein 6 in non-small cell lung cancer and its significance. Oncol Lett.

[139] Takahashi M, Otsuka F, Miyoshi T, Otani H, Goto J, Yamashita M (2008). Bone morphogenetic protein 6 (BMP6) and BMP7 inhibit estrogen-induced proliferation of breast cancer cells by suppressing p38 mitogen-activated protein kinase activation. J Endocrinol.

[140] Kwon SJ, Lee GT, Lee JH, Iwakura Y, Kim WJ, Kim IY (2014). Mechanism of pro-tumorigenic effect of BMP-6: neovascularization involving tumor-associated macrophages and IL-1a. Prostate.

[141] Lee GT, Jung YS, Ha YS, Kim JH, Kim WJ, Kim IY (2013). Bone morphogenetic protein-6 induces castration resistance in prostate cancer cells through tumor infiltrating macrophages. Cancer Sci.

[142] Wan S, Zhao E, Kryczek I, Vatan L, Sadovskaya A, Ludema G (2014). Tumor-associated macrophages produce interleukin 6 and signal via STAT3 to promote expansion of human hepatocellular carcinoma stem cells. Gastroenterology.

[143] He G, Dhar D, Nakagawa H, Font-Burgada J, Ogata H, Jiang Y (2013). Identification of liver cancer progenitors whose malignant progression depends on autocrine IL-6 signaling. Cell.

[144] Jayatilaka H, Tyle P, Chen JJ, Kwak M, Ju J, Kim HJ (2017). Synergistic IL-6 and IL-8 paracrine signalling pathway infers a strategy to inhibit tumour cell migration. Nat Commun.

[145] Zhu J, Fu Q, Wang S, Ren L, Feng W, Wei S (2022). Palladium nanoplate-based IL-6 receptor antagonists ameliorate cancer-related anemia and simultaneously inhibit cancer progression. Nano Lett.

[146] Hailemichael Y, Johnson DH, Abdel-Wahab N, Foo WC, Bentebibel SE, Daher M (2022). Interleukin-6 blockade abrogates immunotherapy toxicity and promotes tumor immunity. Cancer Cell.

[147] Delyon J, Lebbe C (2022). IL-6 blockade in cancer patients treated with immune checkpoint blockade: a win-win strategy. Cancer Cell.

[148] Singh JA, Beg S, Lopez-Olivo MA. Tocilizumab for rheumatoid arthritis. Cochrane Database Syst Rev. 2010;(7):CD008331. 10.1002/14651858.CD008331.pub2 20614469

[149] Neelapu SS, Tummala S, Kebriaei P, Wierda W, Gutierrez C, Locke FL (2018). Chimeric antigen receptor T-cell therapy - assessment and management of toxicities. Nat Rev Clin Oncol.

[150] Kleinegger F, Hofer E, Wodlej C, Golob-Schwarzl N, Birkl-Toeglhofer AM, Stallinger A (2019). Pharmacologic IL-6Ralpha inhibition in cholangiocarcinoma promotes cancer cell growth and survival. Biochim Biophys Acta Mol Basis Dis.

[151] Johnson M, El-Khoueiry A, Hafez N, Lakhani N, Mamdani H, Rodon J (2021). Phase I, first-in-human study of the probody therapeutic CX-2029 in adults with advanced solid tumor malignancies. Clin Cancer Res.

[152] Singh S, Serwer L, DuPage A, Elkins K, Chauhan N, Ravn M (2022). Nonclinical efficacy and safety of CX-2029, an anti-CD71 probody-drug conjugate. Mol Cancer Ther.

[153] Nywening TM, Wang-Gillam A, Sanford DE, Belt BA, Panni RZ, Cusworth BM (2016). Targeting tumour-associated macrophages with CCR2 inhibition in combination with FOLFIRINOX in patients with borderline resectable and locally advanced pancreatic cancer: a single-centre, open-label, dose-finding, non-randomised, phase 1b trial. Lancet Oncol.

[154] Fukuhara H, Ino Y, Todo T (2016). Oncolytic virus therapy: a new era of cancer treatment at dawn. Cancer Sci.

[155] Kurz E, Hirsch CA, Dalton T, Shadaloey SA, Khodadadi-Jamayran A, Miller G (2022). Exercise-induced engagement of the IL-15/IL-15Ralpha axis promotes anti-tumor immunity in pancreatic cancer. Cancer Cell.

